# Cervico-vaginal inflammatory cytokine alterations after intrauterine contraceptive device insertion: A pilot study

**DOI:** 10.1371/journal.pone.0207266

**Published:** 2018-12-04

**Authors:** Priya Sharma, Kamnoosh Shahabi, Rachel Spitzer, Michele Farrugia, Rupert Kaul, Mark Yudin

**Affiliations:** 1 Department of Obstetrics and Gynecology, University of Toronto, Toronto, Ontario, Canada; 2 Department of Immunology, University of Toronto, Toronto, Ontario, Canada; 3 Department of Obstetrics and Gynecology, Mount Sinai Hospital, Toronto, Ontario, Canada; 4 Department of Obstetrics and Gynecology, St. Michael’s Hospital, Toronto, Ontario, Canada; Fred Hutchinson Cancer Research Center, UNITED STATES

## Abstract

In a prospective study of twenty sexually transmitted infection (STI)-free women, we examined the impact of an intrauterine contraceptive device (IUCD) insertion on cervico-vaginal cytokine levels. Nine women chose the levonorgestrel-containing IUCD and eight chose a copper IUCD. A cervico-vaginal swab was collected for cytokine analysis pre-insertion and four weeks post-insertion. Significant increases were noted in levels of IL-1α (median 483.4 versus 316.6 pg/mL, p = 0.046), IL-1β (median 605.7 versus 147.3 pg/mL, p = 0.018), IL-6 (median 570.1 versus 157.3 pg/mL, p = 0.046), TNFα (median 1.19 versus 0.6 pg/mL, p = 0.029) and the chemokine MCP-1 (median 340.2 versus 135.2 pg/mL, p = 0.003). No significant changes were noted in the levels of GM-CSF, IL-8, MIG, MIP-3α, RANTES, IL-10, IL-17, IP-10, MIP-1β. Whether this increase in pro-inflammatory cytokine levels decreases epithelial barrier integrity and enhances susceptibility to STIs, including HIV, merits further study.

## Introduction

The intrauterine contraceptive device (IUCD) is a highly effective and reversible method of contraception, with noncontraceptive benefits, few systemic side effects and rare absolute contraindications to its use. IUCD insertion induces a marked foreign body inflammatory reaction which is largely limited to the uterus, but which can also be measured systemically [1, 2]. Insertion is associated with alterations in cytokine expression within the endometrium [3], and in cross-sectional studies levonorgestrel (LNG) IUCD users had elevated endocervical levels of several inflammatory cytokines [4], while copper IUCD users demonstrated increased GM-CSF levels in cervical mucus [5]. Prospective studies to assess the induction of pro-inflammatory cytokines in cervico-vaginal secretions is important, since elevated levels of these cytokines correlate with enhanced risk of human immunodeficiency virus (HIV) acquisition [6].

One mechanism for the enhanced HIV risk associated with elevated inflammatory cytokines is the recruitment of HIV-susceptible CD4+ T cells. However, while a local leukocytic inflammatory response to an IUCD is seen within the endometrium [7] and fallopian tubes [8], and cervical cellular differences were observed in a cross sectional study of LNG-IUCD users [4], neither copper nor LNG-IUCD insertion altered endometrial and cervical T cell populations in a prospective study [9]. Nonetheless, cervico-vaginal inflammation is also associated with markers of reduced epithelial integrity, suggesting that reduced barrier function may be an independent mechanism of increased HIV risk [10]. Therefore, the primary objective of this study was to prospectively examine the impact of an IUCD on cervico-vaginal cytokine and chemokine levels, to test the hypothesis that IUCD insertion would induce an endocervical proinflammatory cytokine response.

## Methods

Prior to patient enrolment, this study was approved by the Mount Sinai Hospital Research Ethics Board for human subjects research. This was a pilot study, and as such *a priori* power calculations were not used to establish the sample size. Consecutive women requesting IUCD insertion for either contraception or noncontraceptive reasons were eligible to be enrolled from an outpatient Gynecology unit at Mount Sinai Hospital in Toronto, Ontario, and followed prospectively. Participants were excluded if there was a suspected or confirmed pregnancy, history of a chronic inflammatory condition, history of IUD expulsion within the past month, unexplained vaginal bleeding or an inability to provide informed consent. Informed consent was obtained from all study participants prior to enrollment. In order to control for stage of the menstrual cycle, each participant had two study visits, the IUCD insertion visit and a follow-up visit four weeks later. This is part of routine care for women receiving IUCDs at our institution. At the initial visit, a demographic questionnaire was completed. Prior to IUCD insertion, participants were screened for sexually transmitted infections (STIs), including *Chlamydia trachomatis* and *Neisseria gonorrhoeae* by PCR, and *Trichomonas vaginalis* by culture. In addition, testing for bacterial vaginosis by Gram stain and *Candida albicans* by vaginal culture was performed. For cytokine analysis, a cervico-vaginal Copan E-swab was collected and samples were stored in a freezer (-20 °C) in Copan transportation media. The samples were then centrifuged and aliquoted within 48 hours, and stored at -80°C. At the follow up visit four weeks later, both the genital infection screen and cytokine analysis were repeated. Cytokine and chemokine levels were measured using two Human-Ultra-Sensitive custom-made 7-plex electrochemiluminescent based ELISA kits (Meso Scale Discovery, MSD) to measure 14 cytokine/chemokines. The biomarker panel included GM-CSF, IL-1α, IL-8, MCP-1, MIG, MIP-3α, RANTES, IL-10, IL-17, IL-1β, IL-6, IP-10, MIP-1β and TNF-α. The levels of the biomarkers were determined using standard curves (*p*g/ml). Wilcoxon matched-paired analyses were used to analyze intra-individual changes in cytokine levels between the two sampling visits.

## Results

A total of 20 women were enrolled in the study between June 2013 to July 2014. Biomarker data was collected for all 20 participants and demographic information was available for 17 participants. The median age was 30 years old (range 22–39 years old), and other demographic chacteristics are presented in Table 1. Choice of IUCD was at the discretion of the patient and provider. Nine women chose the levonorgestrel-containing IUCD and eight chose a copper IUCD. All participants screened negative for STIs, including *Chlamydia trachomatis*, *Neisseria gonorrhoeae* and *Trichomonas vaginalis* both before and after insertion of the IUCD. In addition, all cultures were negative for *Candida albicans*. One participant was positive by Gram stain for bacterial vaginosis on both the initial screen and follow up visit.

**Table 1:** Demographic characteristics of participants.

Characteristics	N (%)
Race	
Caucasian	12 (70.6)
Black	1 (5.8)
Asian	2 (11.8)
Other	2 (11.8)
Lifetime Male Sexual Partners	
None	1 (5.8)
One	14 (82.3)
More than 5	2 (11.8)
Lifetime Female Sexual Partners	
None	16 (94.1)
One	1 (5.8)
More than 5	0 (0)
History of Bacterial vaginosis	
No	11 (64.7)
Yes	4 (23.5)
Unsure	2 (11.8)
History of STI	
Genital HSV	2 (11.8)
Chlamydia	1 (5.8)

STI—Sexually Transmitted Infection

HSV—Herpes Simplex Virus

**Fig 1:**
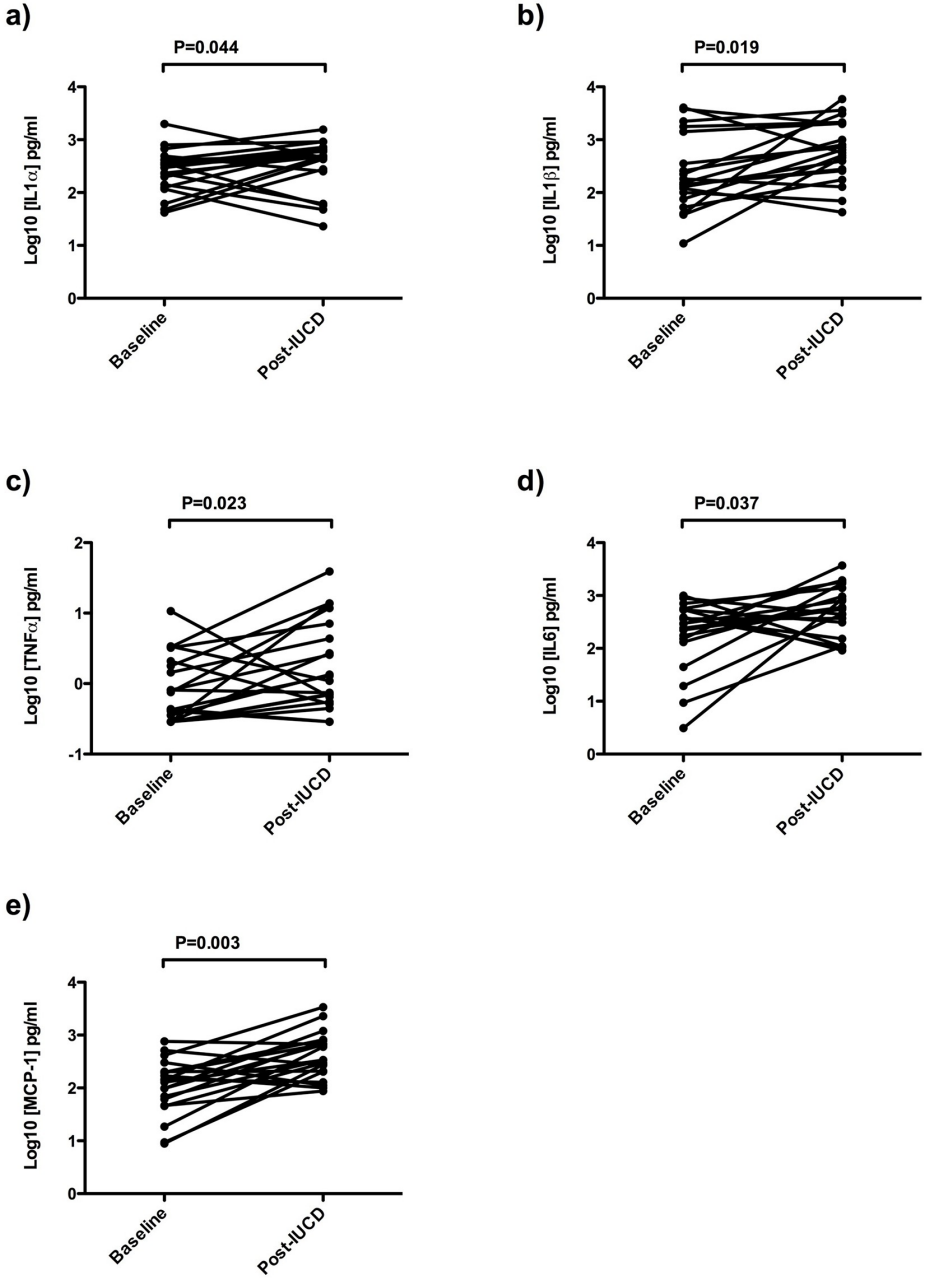
Cervicovaginal cytokine levels before and after IUCD insertion. IUCD–Intrauterine Contraceptive Device Levels of several cytokines were assayed in cervicovaginal samples prior to and one month after IUCD insertion (labeled as ‘Baseline’ and ‘Post-IUCD’ respectively). Lines represent levels over time in each of 20 participants; statistical comparisons used the Wilcoxon test for paired samples.

All cytokines were present at detectable levels in cervical secretions prior to IUCD insertion (Table 2), and several changes were demonstrated in the cervico-vaginal cytokine/chemokine profile after the insertion of an IUCD. Specifically, there was a significant increase in the levels of several classical pro-inflammatory cytokines, including IL-1α (median 483.4 versus 316.6 pg/mL, p = 0.046), IL-1β (median 605.7 versus 147.3 pg/mL, p = 0.018), IL-6 (median 570.1 versus 291.0 pg/mL, p = 0.046) and TNFα (median 1.19 versus 0.6 pg/mL, p = 0.029), as well as the chemokine MCP-1 (median 340.2 versus 135.2 pg/mL, p = 0.003) (Fig 1). No significant differences were seen after IUCD insertion in levels of the cytokines IL-8, MIG, MIP-3α, RANTES, IL-10, IL-1β, IP-10, IL-17 or MIP-1β.

**Table 2:** Baseline cervicovaginal cytokine levels.

**Cytokine**	**Median level (pg/ml)**	**Range (pg/ml)**
**GM-CSF**	1067.9	26.3–10,703.6
**IL-1α**	316.6	41.2–1,985.4
**IL-1β**	147.3	11.1–4,046.1
**IL-8**	3,494.0	328.2–47,057.6
**MCP-1**	135.2	8.8–757.5
**MIG**	28.0	2.7–1034.4
**MIP-3α**	378.5	20.6–6,154.7
**RANTES**	86.7	1.4–4,831.7
**IL-10**	1.9	2.9–131.8
**IL-17**	6.1	2.4–49.8
**IL-6**	291.0	3.1–988.9
**IP-10**	17.6	14.4–1,621.3
**MIP-1β**	504.2	2.4–32,026.6
**TNF**	0.6	0.3–10.8

Despite the relatively small sample size, we observed interesting differences in the cervical cytokines that were induced by different IUCD types. Specifically, insertion of the LNG-IUCD (n = 9) was associated with increased levels of cervical GM-CSF (Wilcoxon paired p = 0.025) and a trend to increased MCP-1 (p = 0.069), while insertion of the copper IUCD (n = 8) induced significant increases in the more prototypic pro-inflammatory cytokines IL-1α (p = 0.05), IL-1β (p = 0.012), IL-6 (p = 0.05) and TNF (p = 0.017), as well as trends to increased MCP-1 and IL-17 (both p<0.1).

## Discussion

This study demonstrated a change in the cytokine profile within the lower genital tract after IUCD insertion, with an increase in several classical pro-inflammatory cytokines. These findings are broadly in keeping with prior cross-sectional studies showing elevated GM-CSF and other cytokines in cervical mucus [4, 5], in addition to elevated cytokines in the endometrial cavity [5, 11]. However, this pilot study employed a prospective design, allowing for intra-individual analysis that can control for potential confounders, and also used a comprehensive biomarker panel to investigate changes in pro-inflammatory and chemoattractant cytokines and chemokines.

Interesting differences were observed between the copper and LNG-IUCD in the induction of cytokines post insertion, with the copper IUCD inducing a more typical mucosal inflammatory cytokine response and the LNG-IUCD inducing GM-CSF. Although a pro-inflammatory reaction exists with both the copper and LNG-IUCD [5,11], and a prior study also observed differences in cytokine profile changes based on the type of IUCD inserted [3], these results should be viewed as hypothesis generating and will need confirmation in future studies.

The cellular and cytokine milieu of the female genital tract mucosa is a determinant of susceptibility to STIs, particularly HIV [6]. In order to find safe options for contraception in patient populations at high risk of acquisition or transmission of HIV, prior studies have focused on the effects of various contraceptive methods on the immunology of the lower genital tract. Neither the quantity of CD4+ T cells nor their expression of CCR5, which constitute important target cells for HIV, were found to be elevated after the insertion of an IUCD [9]. However, the increase in pro-inflammatory cytokines that we observed post-IUCD insertion is similar to that associated with increased HIV susceptibility in South African women [6] and could possibly alter epithelial barrier function and/or be a risk factor for other STIs. Clinically, this may warrant caution in inserting an IUCD in high-risk patients pending further studies regarding the impact on HIV risk, in particular the multinational ECHO randomized trial of contraceptive method and HIV risk [12]

These study results should be framed within a few limitations. Firstly, the types of IUCD inserted were a mix of copper and levonorgestrel-releasing devices, based on patient preference after consultation with the gynecologist. Since the IUCD indication was for contraception purposes in the majority of participants, there was felt to be no advantage of one type over the other. Although there was considerable inter-participant variability in baseline genital cytokine levels, the prospective nature of our study allowed for a paired intra-individual analytic approach, enhancing power by controlling for both known and unknown confounders that may contribute to this heterogeneity. Cyclical changes in reproductive hormones can affect cytokine profiles in the genital tract [13], although this was controlled as much as possible by coordinating participants to return during the early follicular phase of the menstrual cycle. Lastly, genital infections can induce mucosal inflammation and could, if present, confound our study. However, all our patients screened negative for classical STIs, and with the exception of one patient who tested positive for bacterial vaginosis (by Nugent score) before and after insertion of an IUCD, the remaining participants had normal vaginal flora. Therefore, the changes in cytokine profiles were not attributable to newly acquired infections or clinically evident changes in vaginal flora.

In conclusion, we found in this prospective pilot study that insertion of an IUCD resulted in increased levels of multiple classical pro-inflammatory cytokines in cervico-vaginal secretions after four weeks, which were most marked after insertion of the copper IUCD. Some changes were relatively modest, and there was substantial inter-individual heterogeneity in cytokine levels, and so the biological impact and time course of changes seen in this pilot study will need to be elucidated in larger clinical studies. Nonetheless, these findings raise concerns regarding potential enhanced susceptibility to STIs, including HIV, in the immediate post-IUCD insertion period.
